# Full Genome Evolutionary Studies of Wheat Streak Mosaic-Associated Viruses Using High-Throughput Sequencing

**DOI:** 10.3389/fmicb.2021.699078

**Published:** 2021-07-30

**Authors:** Carla Dizon Redila, Savannah Phipps, Shahideh Nouri

**Affiliations:** Department of Plant Pathology, College of Agriculture, Kansas State University, Manhattan, KS, United States

**Keywords:** wheat streak mosaic virus, Triticum mosaic virus, evolutionary studies, high-throughput RNA sequencing, high plains wheat mosaic emaravirus

## Abstract

Wheat streak mosaic (WSM), a viral disease affecting cereals and grasses, causes substantial losses in crop yields. Wheat streak mosaic virus (WSMV) is the main causal agent of the complex, but mixed infections with Triticum mosaic virus (TriMV) and High plains wheat mosaic emaravirus (HPWMoV) were reported as well. Although resistant varieties are effective for the disease control, a WSMV resistance-breaking isolate and several potential resistance-breaking isolates have been reported, suggesting that viral populations are genetically diverse. Previous phylogenetic studies of WSMV were conducted by focusing only on the virus coat protein (CP) sequence, while there is no such study for either TriMV or HPWMoV. Here, we studied the genetic variation and evolutionary mechanisms of natural populations of WSM-associated viruses mainly in Kansas fields and fields in some other parts of the Great Plains using high-throughput RNA sequencing. In total, 28 historic and field samples were used for total RNA sequencing to obtain full genome sequences of WSM-associated viruses. Field survey results showed WSMV as the predominant virus followed by mixed infections of WSMV + TriMV. Phylogenetic analyses of the full genome sequences demonstrated that WSMV Kansas isolates are widely distributed in sub-clades. In contrast, phylogenetic analyses for TriMV isolates showed no significant diversity. Recombination was identified as the major evolutionary force of WSMV and TriMV variation in KS fields, and positive selection was detected in some encoding genomic regions in the genome of both viruses. Furthermore, the full genome sequence of a second Kansas HPWMoV isolate was reported. Here, we also identified previously unknown WSMV isolates in the Great Plains sharing clades and high nucleotide sequence similarities with Central Europe isolates. The findings of this study will provide more insights into the genetic structure of WSM-associated viruses and, in turn, help in improving strategies for disease management.

## Introduction

Wheat (*Triticum aestivum* L.) is one of the leading staple crops in the world. In 2019, the wheat production in Kansas estimated by United States Department of Agriculture’s National Agricultural Statistics Service (USDA NASS) was $1.37 billion (National Agricultural Statistics Service (NASS), 2020). Kansas is the second leading producer of wheat behind North Dakota (National Agricultural Statistics Service (NASS), 2020) in the United States. Unfortunately, viral diseases have a great impact on reducing the yield of wheat globally. In 2017, a viral disease called wheat streak mosaic (WSM) has caused a total of $76 million in yield loss to Kansas farmers (Hollandbeck et al., 2017).

**FIGURE 1:**
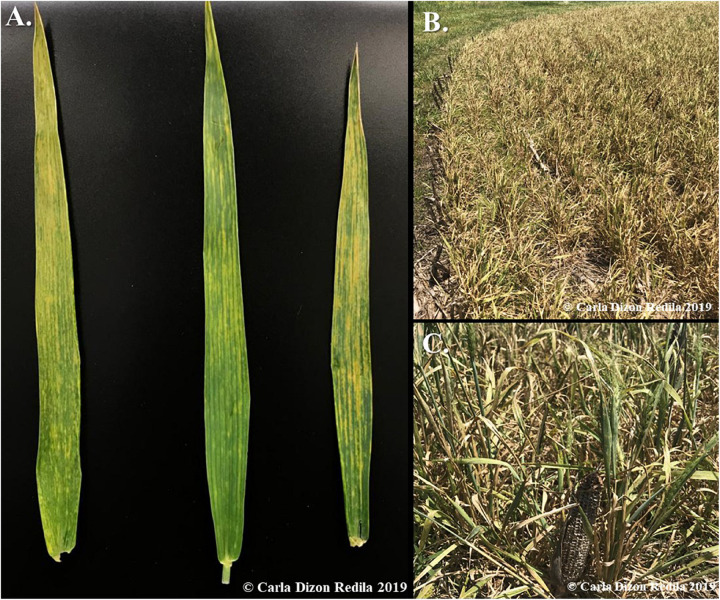
Observed symptoms of WSM in the field. **(A)** Typical viral symptoms of WSM is yellow, mosaic-like streaks on leaves. **(B)** Severe symptoms of mixed infections of WSMV + TriMV include stunting, which leads to the underdevelopment and total loss of the crops. **(C)** A close-up image of the stunted wheat infected with WSM viruses, which is only twice the size of the dried corn cob used for comparison. The expected height of wheat in this ripening stage is three times higher.

Wheat streak mosaic is a disease complex, which consists of three documented viruses: Wheat streak mosaic virus (WSMV), Triticum mosaic virus (TriMV), and High plains wheat mosaic emaravirus (HPWMoV), which are all transmitted by wheat curl mites (WCM), *Aceria tosichella* Kiefer (Slykhuis, 1955; Seifers et al., 1997; Seifers et al., 2009). WSMV and TriMV are type species classified under the *Potyviridae* family and are both filamentous viruses with positive-sense, single-stranded RNA genomes (Stenger et al., 1998; Fellers et al., 2009; Tatineni et al., 2009). In contrast, HPWMoV belongs to the *Fimoviridae* family, which is a multipartite, negative-sense virus consisting of eight RNA segments (Tatineni et al., 2014; Stewart, 2016). The typical symptoms of WSM caused by any of the three viruses in single infections are similar: yellow, mosaic-like streaks on the leaves (Figure 1), which lead to chlorosis and reduction in photosynthetic capabilities. Severe infection may also lead to stunted growth (Figure 1; Singh et al., 2018). For this reason, it is difficult to differentiate the causal virus phenotypically from symptoms, and serological or molecular biology techniques such as ELISA and RT-PCR are required to determine which virus or mixed-infection of viruses is present.

Compared to the other two viruses of the WSM complex, WSMV is the more widely studied and it has a longer history, with its first observation dating back to 1922 in Nebraska (McKinney, 1937). WSMV belongs in the family *Potyviridae* and the genus *Tritimovirus* (Stenger et al., 1998). The genome of WSMV is ∼9.3kb in size and encodes one large polyprotein, which is enzymatically cleaved and forms 10 mature proteins: P1, HC-Pro (helper component protein), P3, 6K1, CI (cytoplasmic inclusion protein), 6K2, NIa-Pro (nuclear inclusion putative protease), NIa-VPg (viral protein genome-linked proteinase), and CP (coat protein) (Choi et al., 2002; Chung et al., 2008; Tatineni et al., 2011; Tatineni and French, 2014, 2016; Singh et al., 2018). The 5′ and the 3′ termini contain a VPg and a Poly (A) tail, respectively (Singh et al., 2018). Previous phylogenetic study based on the coat protein sequence of WSMV divided isolates into four different clades (clades A-D) based on their geographic regions (Stenger et al., 2002; Stenger and French, 2009). The U.S. isolates were placed in clade D and divided into four sub-clades, in which Kansas isolates were distributed throughout the clade (Stenger et al., 2002).

TriMV, a previously unknown wheat virus, was first discovered in Western Kansas in 2006 and its association with WSM was reported (Seifers et al., 2008). TriMV belongs to the family *Potyviridae* like WSMV but different genus, *Poacevirus*: (Fellers et al., 2009; Tatineni et al., 2009). The genome size of TriMV is ∼10.2 kb and, similar to WSMV, encodes a large polyprotein that is cleaved into 10 mature proteins (Fellers et al., 2009). In contrast to WSMV, TriMV has an unusual long 5′ untranslated region (UTR) spanning to 739 nt (Fellers et al., 2009; Tatineni et al., 2009). There are currently no studies demonstrating the phylogeny and genetic variation of TriMV.

HPWMoV, the other documented virus associated with WSM, was first described in 1993 as the causal agent of the high plains disease infecting maize and wheat in the Great Plains (Jensen et al., 1996). However, the genome sequence and organization of HPWMoV were not determined until 2014 (Tatineni et al., 2014). HPWMoV consists of eight negative-sense RNA segments, designated as RNAs 1 to 8 (Tatineni et al., 2014). The encoded proteins are annotated as follows: RNA 1 is the RNA-dependent RNA polymerase (RdRp), RNA 2 is the putative glycoprotein, RNA 3 is the nucleocapsid protein, RNA 4 is the putative movement protein, and RNAs 7 and 8 act as the RNA silencing suppressor (Gupta et al., 2018). RNAs 5 and 6 currently do not have any known functions (Gupta et al., 2018).

To date, three resistant genes against WSM have been identified: *Wsm1* and *Wsm3* against both WSMV and TriMV isolates and *Wsm2* only against WSMV isolates (Triebe et al., 1991; Liu et al., 2011; Lu et al., 2011). In 2019, a WSMV resistant-breaking isolate has been reported and confirmed to overcome *Wsm2* in Kansas (Fellers et al., 2019). Potential resistant-breaking isolates for WSMV and TriMV have also been found to infect resistant varieties in the field (Kumssa et al., 2019). These events place a greater importance in understanding the current genetic structure of natural viral populations associated with WSM in order to determine the distribution of the associated viruses in fields and determine the major evolutionary forces acting upon WSM viruses.

In this study, we determined the current distribution and prevalence of WSM-associated viruses mainly in Kansas fields and some fields in other parts of the Great Plains and assessed the source of genetic variation of the WSM viruses. Additionally, and for the first time, the phylogenetic relationship among WSMV isolates was investigated based on the full genome sequence of historic and field isolates in this study. We also generated the first phylogenetic analysis of TriMV isolates using the whole genome sequence. The complete sequences of eight RNA segments of a new HPWMoV isolate from Kansas were reported as well.

## Materials and Methods

### Wheat Survey and Sample Collection

In 2019, symptomatic and asymptomatic wheat leaf samples were collected through field surveys and sample submissions to the Kansas State University (KSU) Plant Disease Diagnostics Lab (Supplementary Table 1). In addition to these samples, historic WSMV samples were provided by the Agricultural Research Center in Hays, KS (Supplementary Table 1). A few wheat samples from Nebraska and Colorado, other major wheat growing regions in the Great Plains, were also received and included in the study. In 2020, field samples were received from the KSU Plant Disease Diagnostics Lab and wheat samples from Montana were also obtained (Supplementary Table 1).

### Screening Samples for WSM-Associated Viruses

Total RNAs were isolated from leaf tissues using TRIzol reagent (Invitrogen, CA, United States), according to the manufacturer’s instructions. The extracted RNAs from the selected samples were treated with DNase I (Zymo Research, CA, United States). The first strand cDNAs were synthesized using the SuperScript II Reverse Transcriptase (Invitrogen, CA, United States). OligodT or gene specific primers (Supplementary Table 2) were used for the PCR step for each virus. PrimeSTAR GXL Premix (Takara Bio, CA, United States) was used to carry out a 25 μl reaction containing 1× PrimeSTAR GXL Buffer, nuclease-free water, and 0.5 μM each of the gene specific primers. The thermal cycler program used is as follows: 98°C for 2 min, 34 cycles of 98°C for 10 s, 55°C for 15 s, and 68°C for 2 min, and 72°C for 5 min to amplify ∼2 kb products.

For HPWMoV, one step RT-PCR was conducted to screen the samples. For the one step RT-PCR, the 25 μl reactions contained 1× GoTaq Flexi Buffer (Promega, WI, United States), 1 μM MgCl2, 0.1 μM dNTP, 0.4 μM of gene specific primers (Supplementary Table 2), 1.25 U GoTaq Flexi DNA Polymerase (Promega, WI, United States), 200 U of the SuperScript IV Reverse Transcriptase (Invitrogen, CA, United States), 40 U RNaseOUT, and DEPC treated water. The thermal cycler program used is as follows: 42°C for 10 min, 94°C for 2 min, 34 cycles of 94°C for 10 s, 55°C for 30 s, and 72°C for 1 min, and 72°C for 5 min to amplify 500 bp products. The RT-PCR products were visualized on agarose gels stained with SYBR Safe (Invitrogen, CA, United States).

### RNA Library Construction and Sequencing

A total of 20 field sample from 2019 (15 samples) and 2020 (5 samples) were chosen for sequencing based on the results of the virus screenings and the geographic region with mixed infections being prioritized (Supplementary Table 3). Additionally, five historic samples and 1 sample each from Colorado, Montana, and Nebraska were also selected for library preparation (Supplementary Table 3).

The RNA integrity of the DNase treated samples was measured using Qubit 4 (Invitrogen, CA, United States) with the RNA IQ assay kit, according to the manufacturer’s instructions. The quantification of the RNA was carried out using the NanoDrop Spectrophotometer (Invitrogen, CA, United States). The TruSeq Stranded Total RNA with Ribo-Zero Plant Kit (Illumina Inc., CA, United States) was utilized to deplete the rRNA and prepare the libraries for sequencing, following the manufacturer’s instructions. Agencourt RNAClean XP (Beckman Coulter, MA, United States) was used to purify the samples and ensure the removal of all traces of rRNA. TruSeq RNA Single Indexes Sets A and B (Illumina Inc., CA, United States) were used for adapter ligation. After each step of cDNA synthesis, adapter ligation, and enrichment of the DNA fragments, the samples were purified using the Agencourt AMPure XP (Beckman Coulter, MA, United States).

The final libraries were subjected to quality control analysis using Agilent Bioanalyzer 2100 system (Agilent Technologies, CA, United States) and were quantified using the Qubit 4 (Invitrogen, CA, United States) with the 1× dsDNA High Sensitivity Assay (Invitrogen, CA, United States). A total of 22 libraries from 2019 field and historic collections were pooled and sequenced in two lanes (11 pooled libraries in each lane) using the NextSeq 500 (Illumina Inc., CA, United States) high-output with a read length of 1 × 75 bp at the Kansas State Integrated Genomics Facility. From our 2020 collection, a total of six libraries were pooled and sequenced in one lane using the same platform as above.

### Bioinformatics Analysis to Obtain Full Genome Sequences

Libraries were demultiplexed based on the index sequences. Trimmomatic was used to trim the reads for quality, length, and the adapter sequences (Bolger et al., 2014). To ensure the reads no longer contained adapter sequences and were of high quality, FastQC was utilized for quality control (Andrews, 2010). The reference genomes of WSMV, TriMV, and HPWMoV (Supplementary Table 4) were retrieved from GenBank^1^. The trimmed reads were mapped against the reference genomes, and the consensus sequences were extracted using the CLC Genomics Workbench 20 (Qiagen, MD, United States).

### Recombination Analysis

Multiple nucleotide alignments of the consensus sequences from this study (Supplementary Table 6) and the complete reference genome sequences obtained from the GenBank (Supplementary Table 5) were conducted using the MUSCLE alignment in the Geneious Prime 2020.2.4 (Edgar, 2004)^2^. The complete genome sequence alignments were then examined using seven different algorithms integrated in the RDP5 program (Martin et al., 2015). The seven algorithms used are as follows: RDP (Martin and Rybicki, 2000), GENECONV (Padidam et al., 1999), MaxChi (Maynard Smith, 1992), BootScan (Martin et al., 2005), Chimaera (Posada and Crandall, 2001), 3SEQ (Lam et al., 2018), and SiScan (Gibbs et al., 2000). The recombination events that were significant (p < 0.01) for at least four out of the seven detection methods were considered as putative recombinants, and potential parents were determined.

The results from RDP5 were utilized to run Bootscan (Salminen et al., 1995) analysis in the SimPlot program (Lole et al., 1999) in order to verify the recombination events. The potential recombinants obtained from the RDP5 program were utilized as query sequences. To run this analysis, sequences of the major and minor parents detected by the RDP5 along with two selected reference sequences were used following the default settings for window width of 200 and step size of 20. The cutoff value for the percent of permuted trees to accept the sample as a potential recombinant was set at 70%. The basic principle of bootscanning is that high levels of phylogenetic relatedness between query and reference sequences found in different regions of the genome may be due to “mosaicism” (Salminen et al., 1995). In addition to Bootscan, SimPlot analysis (Lole et al., 1999) was also utilized using default parameters in order to determine the recombination breakpoints. For this analysis, the major and minor parents were used as references and the recombinant as the query sequence. The crossover points of each reference sequence were deemed as the recombination breakpoint sites.

### Phylogenetic Analysis

The putative recombinants were removed, and outgroups were added before realignment with MUSCLE (Edgar, 2004). The best fitting nucleotide substitution models were determined by the jModelTest 2 (Guindon and Gascuel, 2003; Darriba et al., 2012). The nucleotide substitution models selected by both the Akaike information criterion (AIC) and Bayesian information criterion (BIC) to construct the phylogenetic trees were GTR + I + G for WSMV and GTR + I for TriMV (Guindon and Gascuel, 2003; Darriba et al., 2012). For the phylogenetic analysis, mrBayes plugin within the Geneious Prime 2020.2.4 program was used to construct Bayesian consensus phylogenetic trees using the following default parameters: heated chains of 4, heated chain temp of 0.2, burn in length of 100,000, and sampling every 200 for every 1,100,00 generations (Huelsenbeck and Ronquist, 2001; Ronquist and Huelsenbeck, 2003).

### Population Genetics Analysis

To conduct the population genetics analyses, only the complete genome sequences of Kansas isolates were utilized. Twenty-six isolates for WSMV and 10 isolates for TriMV (Supplementary Table 6) were analyzed using the DnaSP version 6 (Rozas et al., 2003) to calculate the population genetics parameters and genetic diversity.

### Neutrality Tests

The estimation of non-synonymous substitutions (dN), synonymous substitutions (dS), and their ratio (dN/dS = ω) was calculated in MEGA 5 by using the bootstrap method with 1000 replicates under the model of the Kumar method for each encoded protein (Kumar et al., 2004; Tamura et al., 2011). Using Hyphy 2.2.4 (Pond et al., 2005), the stop codons for the polyprotein alignments of the Kansas isolates were removed prior to neutrality tests. To evaluate the selection pressure by site of specific codons, three different methods that are implemented in the Hyphy package were used (Pond et al., 2005). Fixed effects likelihood (FEL) and single likelihood ancestor counting (SLAC) utilize the maximum-likelihood (ML) methods to analyze site specific selection pressures of the polyprotein (Kosakovsky Pond and Frost, 2005). In addition to the ML methods, a Bayesian approach using fast, unconstrained Bayesian approximation (FUBAR) was also applied (Murrell et al., 2013). The default cutoff P-value set by Hyphy of 0.1 for SLAC and FEL and 0.9 of Bayes Factor for FUBAR were utilized to determine the significance of the results. The codons determined to be significant by at least two methods were accepted as the sites under positive or negative selections.

## Results

### WSM Distribution in Kansas

**FIGURE 2:**
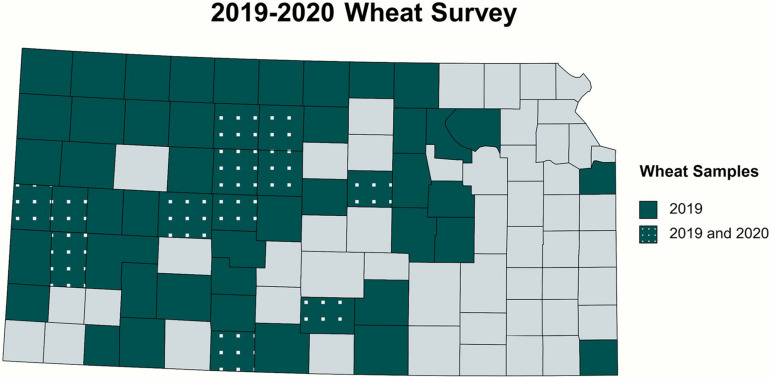
Collection sites of the wheat samples surveyed in 2019 and 2020. Symptomatic and asymptomatic leaf samples were screened for WSM-associated viruses. The survey covered 54 counties from central and western Kansas and two eastern counties.

**FIGURE 3:**
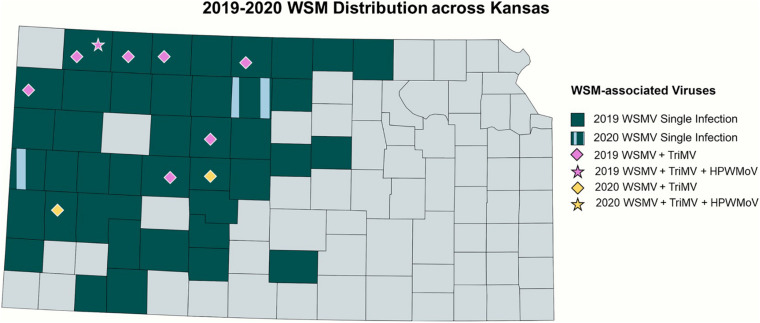
Distribution of WSM-associated viruses in Kansas. 2019 single infections of WSMV is highlighted in dark blue, and single infections of WSMV in both 2019 and 2020 were highlighted with vertical patterns of light blue. The mixed infections of WSMV + TriMV are depicted by the diamonds, and the mixed infection of WSMV + TriMV + HPWMoV is shown in the star, with the purple color depicting the 2019 isolates and yellow for 2020.

In total, 84 and 14 field-collected leaf samples from 2019 and 2020, respectively, were screened for WSM-associated viruses by RT-PCR. Figure 2 demonstrates the collection sites of the surveyed wheat samples^3^. The number of the collected samples was smaller in 2020 because of the COVID-19 pandemic and research restrictions. Sample screening for WSM viruses using RT-PCR revealed that single infections of WSMV dominated Kansas fields at 52% (44 positive out of 84 samples) and 29% (4 positive out of 14 samples) in 2019 and 2020, respectively (Supplementary Table 1). This was followed by 8% (7 positive out of 84 samples) and 14% (2 positive out of 14 samples) of mixed infections of WSMV + TriMV in 2019 and 2020, respectively (Supplementary Table 1). A mixed infection of WSMV + TriMV + HPWMoV was detected in only one sample of 2019 and none in 2020. Figure 3 illustrates the distribution of WSM-associated viruses across the state of Kansas. No single TriMV or HPWMoV infections were detected in either years(see text footnote 3). Only WSMV was detected in historic samples as well as three samples from Nebraska, Colorado, and Montana (Supplementary Table 1).

### RNASeq Analysis Obtained Full Genome Sequences of WSM Viruses

In total, the average of 33 million reads were produced for each library from total RNA sequencing (Supplementary Table 3). All raw sequences were deposited in the GenBank under the BioProject number PRJNA722004. After trimming, an average of 26 million clean reads were obtained which were used for mapping against reference viral sequences. Over 95% of the complete genome sequences of 21 WSMV, 9 TriMV, and 1 HPWMoV from field collected samples and 5 historical WSMV samples were obtained by mapping (Supplementary Tables 5, 9). Complete genome sequences were used for the rest of analyses. Two samples infected with WSMV, NT19, and KE19, did not produce enough coverage to obtain full genome sequences and were excluded from the WSMV studies. Although full genome sequences of all eight RNA segments of a HPWMoV isolate were obtained in this study (Supplementary Table 10), we did not perform the phylogenetic or population genetics analyses for HPWMoV in this study for two reasons: First, the prevalence of HPWMoV was very low in our survey (only one infected sample), and second, there were only five complete genome sequences of HPWMoV available in the GenBank at the time of preparation of this report.

### Full Genome Sequence Alignment of WSM Viruses

#### WSMV

The full genome sequences were aligned with sequences obtained from the GenBank. Most of the WSMV isolates from Kansas exhibited high nucleotide similarity (≥95%) with other isolates from the U.S. and lower similarity (≥ 88%) with Central Europe and Iran isolates. However, two isolates from Kansas (KM19 and RO20) and one isolate from Nebraska (NE01_19) showed higher similarity (∼94%) with Central Europe isolates and lower similarity (∼89%) with isolates from other U.S. states.

#### TriMV

The full genome sequence alignment of the TriMV isolates obtained in this study with available complete genome sequences retrieved from the GenBank including Nebraska, Colorado, and other Kansas isolates revealed high sequence similarities (≥98) between isolates.

#### HPWMoV

Complete genome sequences of eight segments of a HPWMoV isolate from Kansas obtained in this study (Supplementary Table 10) were aligned individually with reference genomes from the GenBank, resulting in high similarities (≤ 95%) of all segments with isolates from Nebraska, Kansas, Michigan, and one isolate from Ohio (GG1). In comparison, three other isolates from Ohio (NW1, NW2, and W1) showed lower similarities (∼60%–70%) to the isolate obtained in this study. These results suggest that HPWMoV U.S. field isolates are genetically diverse. However, a greater number of isolates should be studied in future to gain a better understanding of the genetic diversity of natural populations of HPWMoV.

### Recombination Analysis

#### WSMV

The WSMV recombination analysis consisted of full genome sequences of 15 field samples collected in 2019, 6 field samples in 2020, 5 historical samples (Supplementary Table 6), and complete reference sequences (Supplementary Table 5) retrieved from the GenBank. In total, 11 potential recombinants (42%) were identified for WSMV. DC19, KM19, KSH294, EL17-1183, NE01-19, NS02-19, RO20, RH20, COPhil, SM19, and KM19 isolates were detected as putative recombinants by at least five RDP5 detection methods with a significant support (p < 0.05; Supplementary Table 7). The detection methods also identified the potential parents, which included isolates from Nebraska, Washington, Czech Republic, Kansas, and Poland (Supplementary Table 8). The putative recombinants were confirmed using the BootScan method (Supplementary Figure 1) and SimPlot (Supplementary Figure 2). Recombination hotspots were detected in the regions of WSMV genome encoding HC-Pro, P3, NIb, CI, NIa-VPG, and P1 proteins (Supplementary Figure 2).

#### TriMV

For the recombination analysis of TriMV, genome sequences of seven field samples from 2019, two isolates from 2020 (Supplementary Table 6), and complete genomes obtained from the GenBank (Supplementary Table 5) were used. The RDP5 program found one significant putative recombinant (11%) in the 2020 isolate, RH20 (Supplementary Table 9), and it was confirmed using the BootScan method (Supplementary Figure 3). The recombination breakpoint sites were found in the NIa-Pro, CI, NIb, and 5′ UTR of the genome using the SimPlot method (Supplementary Figure 4).

### Phylogenetic Analysis

#### WSMV

**FIGURE 4:**
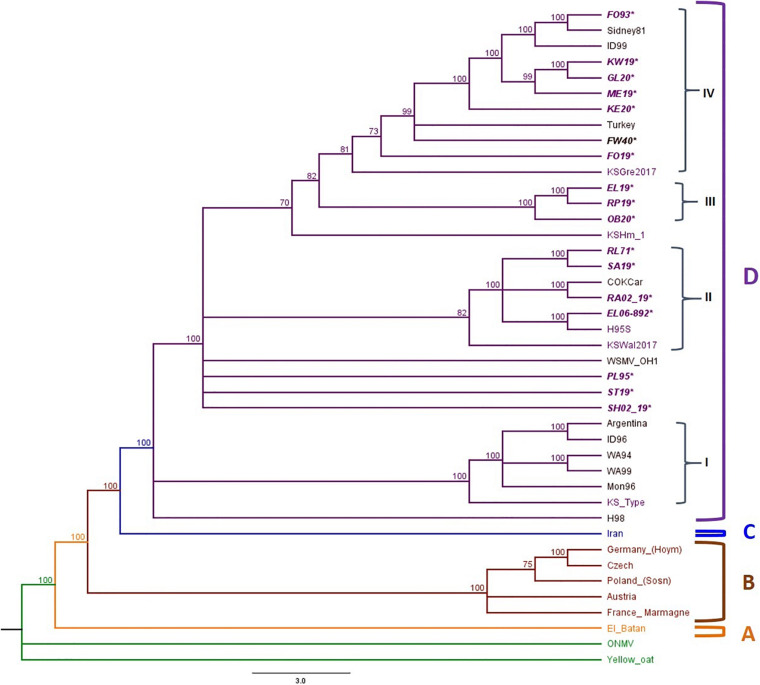
Phylogenetic tree of WSMV isolates is divided into Clades A to C and Clade D is further divided into four sub-clades: I–IV. The sample IDs of Kansas isolates are in purple text, and the isolates sequenced in this study are written in bold and italicized text. The posterior probability of 70% was the cutoff value, and branches not supported were collapsed.

All putative recombinants were removed prior to phylogenetic analysis. The complete genome sequences of 10 WSMV isolates from 2019 field surveys, 3 field isolates from 2020, 4 WSMV historical samples, and reference isolates obtained from the GenBank (Supplementary Table 5) were used to build the phylogenetic tree. Oat necrotic mottle virus (ONMV) and Yellow oat-grass mosaic virus (YOgMV) were chosen as outgroups. The WSMV topology consists of four main clades: Clade A: an isolate from Mexico, Clade B: European isolates, Clade C: an isolate from Iran, and Clade D: United States, Argentina, and Turkey isolates (Figure 4). Clade D was further divided into four sub-clades (D1–D4), with Kansas isolates widely distributed in all sub-clades (Figure 4).

Sub-clade D1 contained isolates from the American Pacific Northwest (APNW) and the Kansas Type isolate, a previously reported isolate from Kansas (Robinson and Murray, 2013). Sub-cade D2 consisted of one isolate from Colorado, one potential resistant breaking isolate from Kansas, and both historical and 2019 isolates from Kansas. Sub-clade D3 included only Kansas isolates from this study. Sub-clade D4 included isolates from Nebraska, Colorado, Turkey, Idaho, and Kansas. There are polytomies and a small group found within sub-clade 4, which form clusters of isolates from Nebraska with Idaho and previously reported Kansas isolates and another cluster of Kansas isolates from this study.

#### TriMV

**FIGURE 5:**
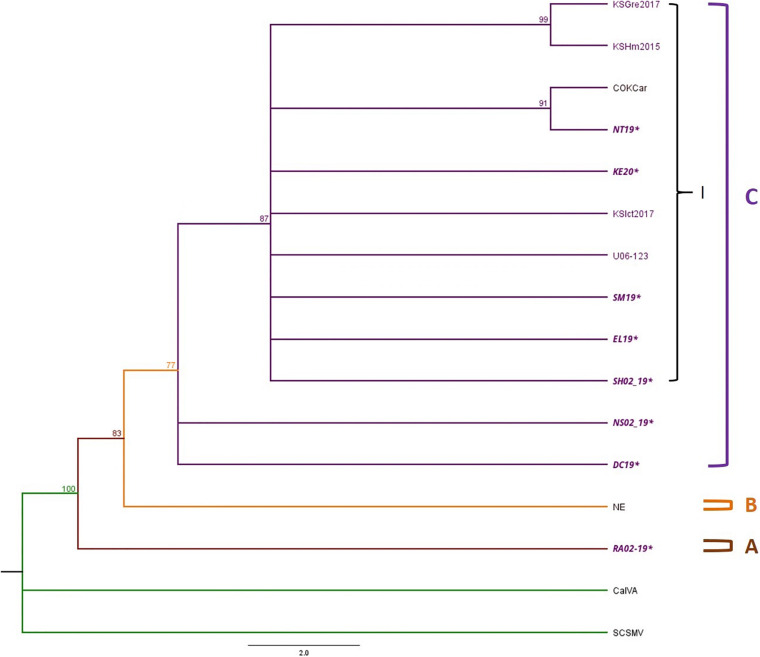
Phylogenetic tree of TriMV isolates is divided into Clades A–C and Clade C contains sub-clade I. The Bayesian phylogenetic tree of the complete genome sequences of TriMV consisted of eight field isolates 2019 and 2020 and six isolates obtained from the GenBank. The sample IDs of Kansas isolates are written in purple text, and the isolates sequenced in this study are written in bold and italicized text. The posterior probability of 70% was the cutoff value, and branches not supported were collapsed.

Phylogenetic trees were constructed with 12 TriMV isolates, including 9 isolates from this study and 6 complete genome sequences available in GenBank (Supplementary Tables 5, 6). Sugarcane mosaic virus (ScSMV) and Caladenia virus A (CalVA) were used as outgroups for constructing the TriMV Bayesian tree (Supplementary Table 5). The topology of TriMV tree consisted of three clades (Figure 5). Clade A included a single isolate from 2019 KS isolate, RA02_19, which was an isolate found in the triple infection of WSM. Clade B contained an isolate from Nebraska. Clade C consisted of one sub-clade (C1) and two polytomies including two isolates from 2019 KS field collection: DC19 and NS02_19. The sub-clade C1 contained five Kansas isolates from this study, one reference isolate from 2016, one isolate from Colorado, and three previously reported potential resistant-breaking isolates from Kansas: KSGre2017, KSHm2015, and KSIct2017 (Fellers et al., 2019). Two of these potential resistant-breaking isolates are closely related and formed their own sister taxa. In addition to this, the Colorado isolate also forms a sister taxa group with a Kansas isolate from 2019 (Figure 5).

### Population Genetics Parameters

The full genome sequences of a total of 26 and 10 Kansas isolates were used to evaluate the genetic diversity of the WSMV and TriMV, respectively (Supplementary Table 6). The population genetic parameters including the average nucleotide diversity (π) and the mutation rate per segregating sites (θw) were calculated for both WSMV and TriMV using DnaSP6 (Tables 1, 2). The nucleotide diversity for both WSMV and TriMV isolates was relatively low with a mean of 0.035 and 0.0039, respectively.

**TABLE 1:** Population genetics parameters calculated using DnaSP and MEGA for encoded regions of Kansas WSMV isolates.

Genomic region	Number of isolates	^1^S	^2^η	^3^π	^4^θ_*W*_	^5^dS	^6^dN	^7^dN/dS (ω)
P1	26	216	231	0.035 ± 0.007	0.053	0.12 ± 0.01	0.0068 ± 0.001	0.057
HC-Pro	26	268	297	0.044 ± 0.009	0.061	0.17 ± 0.01	0.0066 ± 0.001	0.039
P3	26	113	126	0.024 ± 0.005	0.036	0.082 ± 0.009	0.0022 ± 0.0007	0.027
6K1	26	29	32	0.036 ± 0.007	0.050	0.13 ± 0.031	0.0020 ± 0.002	0.015
CI	26	417	468	0.045 ± 0.007	0.057	0.18 ± 0.009	0.002 ± 0.0004	0.011
6K2	26	29	32	0.037 ± 0.007	0.050	0.20 ± 0.03	0.00 ± 0.00	0.000
NIa-VPg	26	131	136	0.040 ± 0.008	0.060	0.16 ± 0.01	0.0015 ± 0.0007	0.0093
NIa-Pro	26	1469	159	0.030 ± 0.00	0.057	0.10 ± 0.009	0.0023 ± 0.0008	0.023
NIb	26	352	383	0.035 ± 0.008	0.062	0.13 ± 0.008	0.0046 ± 0.001	0.035
CP	26	187	201	0.026 ± 0.005	0.047	0.077 ± 0.006	0.0062 ± 0.001	0.081

*^1^Total number of segregating sites. ^2^Total number of mutations. ^3^Nucleotide diversity with the standard deviation calculated by DnaSP. ^4^Estimate mutation rate using segregating sites. ^5^Number of synonymous substitutions per site from the overall mean of sequence pairs. ^6^Number of non-synonymous substitutions per site from the overall mean of sequence pairs. ^7^Ratio of dN/dS used to determine the selective pressure for coding regions.*

**TABLE 2:** Population genetics parameters calculated using DnaSP and MEGA for encoded regions of TriMV.

Genomic region	Number of isolates	^1^S	^2^η	^3^π	^4^θ_*W*_	^5^dS	^6^dN	^7^dN/dS (ω)
P1	10	30	31	0.0055 ± 0.0009	0.0092	0.010 ± 0.002	0.0025 ± 0.001	0.25
HC-Pro	10	29	29	0.0046 ± 0.0008	0.0073	0.013 ± 0.003	0.00090 ± 0.0005	0.069
P3	10	17	18	0.0040 ± 0.0008	0.0067	0.0081 ± 0.002	0.0015 ± 0.0008	0.19
6K1	10	1	1	0.0012 ± 0.001	0.0021	0.0035 ± 0.004	0.00 ± 0.0	0.00
CI	10	38	39	0.0040 ± 0.0005	0.0069	0.011 ± 0.002	0.00084 ± 0.0004	0.076
6K2	10	1	1	0.0013 ± 0.001	0.0023	0.0040 ± 0.004	0.00 ± 0.0	0.00
NIa VPg	10	11	11	0.0037 ± 0.0006	0.0065	0.005 ± 0.001	0.0 ± 0.0	0.00
NIa Pro	10	16	16	0.0052 ± 0.001	0.0083	0.0079 ± 0.002	0.0 ± 0.0	0.00
NIb	10	44	44	0.0061 ± 0.001	0.011	0.017 ± 0.003	0.0019 ± 0.0007	0.11
CP	10	12	12	0.0031 ± 0.0006	0.0048	0.0067 ± 0.002	0.0015 ± 0.001	0.22

*^1^Total number of segregating sites. ^2^Total number of mutations. ^3^Nucleotide diversity with the standard deviation calculated by DnaSP. ^4^Estimate mutation rate using segregating sites. ^5^Number of synonymous substitutions per site from the overall mean of sequence pairs. ^6^Number of non-synonymous substitutions per site from the overall mean of sequence pairs. ^7^Ratio of dN/dS used to determine the selective pressure for coding regions.*

#### WSMV

For WSMV, the CI gene showed the highest diversity (π = 0.045), while the CP possessed the lowest diversity (π = 0.026) (Table 1). The order of the average of nucleotide diversity for all encoded regions of WSMV was as follows: CI > HC-Pro > NIa-VPG > 6K2 > 6K1 > P1 > NIb > NIa-Pro > CP > P3. The degrees of tolerance for amino acid changes (dN/dS) were also calculated for each encoded region, with the 6K2 and the CP as the most and the least tolerant regions, respectively (6K2 > NIa-VPG > CI > 6K1 > NIa-Pro > P3 > NIb > HC-Pro > P1 > CP).

#### TriMV

For TriMV, the NIb and 6K1 genes possessed the greatest (π = 0.0061) and the lowest diversity (π = 0.0012), respectively (Table 2). In contrast to WSMV, the encoded regions with higher diversity for TriMV (NIb > P1 > NIa-PRO > HC-Pro > P3 > CI > NIa-VPG > CP > 6K2 > 6K1) contained greater tolerance for amino acid changes (6K1 > 6k2 > NIa-VPG > NIa-Pro > HC-Pro > CI > NIb > P3 > CP > P1).

### Neutrality Tests

The ratio of dN/dS for all individual proteins was < 1 for both WSMV and TriMV isolates, suggesting purifying (negative) selection as the main selection pressure acting upon encoded proteins. To assess the selection imposed on each site (codon) of individual proteins, three different algorithms were used. Although most sites were detected under the negative selection (data not shown), sites 118 (D→N) and 2525 (G→E) located in the P1 and the NIb proteins of WSMV, respectively, were positively selected sites by two algorithms (Table 3 and Supplementary Figures 5, 6). Codon changes were detected in both historical and field isolates (Supplementary Figures 5, 6). Moreover, site 2677 (L→I) located at the NIb protein of TriMV was detected as a positively selected site as well (Table 3 and Supplementary Figure 7). This change was observed in only one TriMV isolate (RA02_19) (Supplementary Figure 7). All sites were supported significantly by two methods, FEL (p < 0.1) and FUBAR, with the Bayes posterior probability above 0.90 (Table 3).

**TABLE 3:** Codon positions of the coding regions in WSMV and TriMV isolates affected by positive selection.

Virus	Site	^1^FEL dN-dS	FEL *P*-value	SLAC dN-dS	SLAC *P*-value	^1^FUBAR dN-dS	Bayes posterior probability
WSMV	118	5.12	0.048	11.60	0.18	16.76	0.99
WSMV	2525	3.5	0.077	8.80	0.19	9.2	0.97
TriMV	2677	16.66	0.076	84.64	0.19	29.62	0.94

*^1^These methods produced significant results.*

## Discussion

Our study found WSMV as the most prevalent WSM-associated virus in Kansas fields in single infections followed by mixed infections of WSMV + TriMV. This result is consistent with previous surveys (Byamukama et al., 2013). While the findings of the previous studies demonstrated 91% of the TriMV infections in mixed with WSMV, our study did not find any single TriMV infections but all (100%) in mixed with WSMV. To interpret this result, we should know about the distribution of WSM in the field, which relies heavily on the successful transmission of the viruses by WCM. Transmission efficiency studies of WCM for both single and mixed infections found WCM to be efficient in transmitting WSMV alone, whereas TriMV had to be in a mixed infection to increase the transmission efficiency (Seifers et al., 2009). In another study focusing on HPWMoV transmission in a single infection, WCM from Kansas was shown to vector single infections of HPWMoV poorly in comparison to WSMV (Seifers et al., 2002). In addition to the variation in vector transmission for each virus, the transmission efficiency of different biotypes of WCM is also different, in which Biotype 1 has a lower rate of virus transmission efficiency than biotype 2 (Oliveira-Hofman et al., 2015). Biotype 1 has been also shown to be a very poor vector of HPWMoV and could not transmit TriMV in single infections (Seifers et al., 2002; McMechan et al., 2014). Furthermore, it has been shown that the presence of WSMV in mixed infections led to increased efficiency of transmission of HPWMoV and TriMV by WCM (Seifers et al., 2002; Oliveira-Hofman et al., 2015). The presence of both WCM biotypes in Kansas wheat fields and the transmission of WSM viruses by different biotypes with the fitness advantage brought forth by mixed infections may explain the distribution of WSM associated viruses in this study and previous surveys. In addition to this, a mixed infection of WSMV and TriMV was observed to increase the titer of TriMV into the later stages of infection, which may explain the high occurrence of TriMV in mixed compared to single infections (Tatineni et al., 2019).

Our evolutionary analysis suggested recombination as the major evolutionary force operated upon field WSMV (42%) and TriMV (11%) populations. To the best of our knowledge, this is the first comprehensive analysis using full genome sequences to determine the major evolutionary mechanism of WSMV and TriMV variations. The identified potential major and minor parents for WSMV putative recombinants suggested traces of isolates from other U.S. regions as well as countries from Central Europe in Kansas fields (Supplementary Table 8). Interestingly, two putative recombinants from Kansas and one from Nebraska were found in the same clade with Central Europe isolates with a higher nucleotide similarity compared to the rest of the U.S. isolates, suggesting close phylogenetic relationship between these isolates (Supplementary Figure 8). To increase our confidence about this result, we compared the sequence of the CP region of these three putative recombinants with the European isolates, and we observed that two of the three putative recombinants KM19 and NE01_19 from Kansas and Nebraska, respectively, contain three nucleotide deletions in the CP region corresponding to the Gly_2761_ codon (data not shown), which is a characteristic found in all European WSMV isolates (Gadiou et al., 2009). Taking this into account, this is the first report of European WSMV isolates found in the Great Plains fields, along with putative recombinants containing traces of these isolates in their genome. It still remains unclear about whether or not these European isolates were brought to the Great Plains directly from seed exchange with Central Europe or indirectly from the APNW, which first reported the presence of WSMV Central Europe isolates in the U.S. (Robinson and Murray, 2013).

While previous studies, which only focused on the sequence of the CP of WSMV, reported the 3′ terminus of the CP region as the recombination hotspot (Robinson and Murray, 2013), our analysis detected more hotspots in other regions of WSMV genome in addition to the CP. Unlike WSMV, only one recombination breakpoint site in TriMV was found in the 5′ UTR, which has been found to contain a translation enhancing element (Roberts et al., 2015). It is worth noting that the number of the studied TriMV isolates here was lower than WSMV because of the less incidence of TriMV in fields. Therefore, the analysis of a larger number of TriMV isolates would be needed for more accurate assessment of the recombination rate in the field. Overall, our full genome evolutionary analyses indicate that due to different functions of encoded proteins and the variability of evolutionary pressures placed upon them to increase fitness, it is crucial to conduct whole genome analyses of viruses in order to provide a thorough evolutionary study.

Furthermore, our phylogenetic analysis of WSMV using the full genome sequences placed the U.S. isolates in Clade D, which is further divided into four sub-clades. This is consistent with the previous grouping of WSMV isolates based on the CP sequence (Rabenstein et al., 2002; Stenger et al., 2002; Stenger and French, 2009). The widespread distribution of WSMV KS isolates within sub-clades in clade D suggests that although KS isolates are closely related, there is enough diversity to group these isolates in separate clusters. The grouping of historical WSMV KS isolates with 2019 and 2020 isolates in sub-clades 2 and 4 demonstrates that the genetic structure of WSMV populations in the field has not significantly changed from past to present. However, a contrasting observation was found in sub-clade 3 and the cluster within sub-clade 4, which contained isolates only from 2019 and 2020. The opposing observations of the relationship between historical and current KS isolates show the diversity of the viral populations in KS fields and poses a problem with determining the most dominant isolate found in the field.

This study also presented the first recorded phylogenetic study of TriMV. Before the current study, the complete genome sequences of only six TriMV isolates were deposited in the GenBank, in which four of them were from Kansas (Fellers et al., 2009, 2019). Through this study, we were able to generate the full genome sequences of nine TriMV isolates from Kansas fields. Although the genetic variation of TriMV isolates was low, the phylogenetic relationship of these isolates provided an insight into the potential evolutionary pressures, which may be acting upon this virus. Interestingly, some of our 2019 field isolates were grouped together with recently reported potential resistant-breaking isolates with a high support (bootstrap value of 99%) along with a 2019 isolate (NT19) forming a sister taxa with a recently reported isolate from Colorado. This close relationship suggests that natural populations of TriMV may be under pressure to evolve due to the widely use of resistant wheat varieties in the field. However, the analysis of a greater number of TriMV isolates would be needed to validate that claim.

Overall, we observed low genetic diversity for both WSMV and TriMV natural populations. This observation is not surprising, as similar results have previously been reported for populations of this virus and most plant viruses, such as Wheat yellow mosaic virus, Cucumber mosaic virus, and Citrus psorosis virus (Stenger et al., 2002; Martín et al., 2006; Sun et al., 2013; Nouri et al., 2014). In fact, our findings are consistent with the concept that genetic stability is the rule in natural plant virus populations (García-Arenal et al., 2001). Genetic bottleneck during the cell to cell movement and vector transmission have been suggested to be possible reasons for the reduction in variation of WSMV (French and Stenger, 2003).

In addition, purifying selection may also be aiding in reducing diversity and maintaining a genetic stability in plant viruses. Purifying selection was found as the main selection pressure acting on the whole encoding regions of WSMV and TriMV genomes as shown in the ratio of dN/dS < 1 in this study (Tables 1, 2), which is consistent with the previous studies of WSMV focusing only on the CP encoding genomic region (Choi et al., 2001; Stenger et al., 2002; Robinson and Murray, 2013). However, our comprehensive neutrality tests using the polyprotein sequences found a few positively selected sites (codons) for both WSMV and TriMV (Table 3). To the best of our knowledge, this is the first deep analysis of the natural selections imposed on every single codons of the encoded proteins of these two viruses. Through positive selection, viruses may introduce changes to successfully expand host range or host and vector adaptation (Nigam et al., 2019). The two positive selection sites in WSMV were identified at the P1 and NIb regions, and for TriMV, the positively selected site was found in the NIb region. To date, no functional analysis has been performed for any of the codons associated with these specific sites. The P1 protein is the main silencing suppressor of WSMV, and its role as the pathogenicity enhancer has also been determined (Tatineni et al., 2012; Young et al., 2012). The NIb protein is the replicase, which aids in viral replication (Tatineni et al., 2009). The NIb is highly conserved among potyviruses and is often under strong purifying selection; however, mutations in the NIb were found in some potyviruses in order to adapt to different hosts (Stenger et al., 1998; Nigam et al., 2019; Shen et al., 2020). Hence, it is likely that sites 118 and 2525 have undergone evolutionary changes to successfully counter plant and/or vector defense, and also increase pathogenicity in order to adapt to infection of the different wheat varieties, WCM biotypes, and moving from the wheat to other grasses serving as the green bridge (Singh et al., 2018).

On the other hand, competitive replication may likely also pressure TriMV to introduce changes in the NIb region due to the high occurrence of mixed infection with WSMV. The NIb is also essential in interactions with viral and host proteins, leading to formation of viral replication complexes (VRCs) and also post translation, and targets plant defense pathway proteins to suppress immunity response, in addition to serving as the RdRp (Shen et al., 2020). The only TriMV isolate found in this study to have the amino acid changes in the NIb region was RA02_19, which was an isolate containing a triple infection. We hypothesize that the presence of WSMV and HPWMoV may lead into competition with TriMV in recruiting host proteins to form VRCs or trigger immune responses from the host, leading to the introduction of changes in the NIb protein. An improved understanding of the function of these regions and codons for both viruses remains as an interesting aspect that warrants further investigation.

The full genome sequences of eight segments of a HPWMoV isolate were also reported here, which based on our knowledge is the second Kansas isolate to be completely sequenced to date, but compared to the first Kansas isolate, it was isolated from wheat (Supplementary Table 10). In previous studies, the RNA 3 of HPWMoV was found to contain two variants: RNAs 3A and 3B (Tatineni et al., 2014; Stewart, 2016). The Kansas HPWMoV isolate from this study also contained both variants of RNA 3 (data not shown).

Taken together, the results obtained from this study demonstrated the importance of the whole genome sequence analyses to produce more informative data to study field populations of the WSM-associated viruses. Gaining a better understanding of the genetic variation and evolutionary mechanisms utilized by WSMV, TriMV, and HPWMoV natural populations would aid in creating more effective and durable disease management strategies, and help identifying key evolutionary mechanisms utilized by the viruses to overcome current resistance to successfully infect the wheat.

## Data Availability Statement

The raw datasets presented in this study can be found in NCBI BioProject: PRJNA722004. The accession number(s) can be found in the Supplementary Material.

## Author Contributions

SN formulated and designed the experiments. CR and SP processed the samples and performed the experiments. CR conducted the bioinformatics and data analyses. SN and CR wrote and edited the manuscript. All authors contributed to the article and approved the submitted version.

## Conflict of Interest

The authors declare that the research was conducted in the absence of any commercial or financial relationships that could be construed as a potential conflict of interest.

## Publisher’s Note

All claims expressed in this article are solely those of the authors and do not necessarily represent those of their affiliated organizations, or those of the publisher, the editors and the reviewers. Any product that may be evaluated in this article, or claim that may be made by its manufacturer, is not guaranteed or endorsed by the publisher.

## Footnotes

1
www.ncbi.nlm.nih.gov/genbank/

2
www.geneious.com

3
www.mapchart.net
