# Supplementary material for: Full Genome Evolutionary Studies of Wheat Streak Mosaic-Associated Viruses Using High-Throughput Sequencing
Source: Front Microbiol. 2021 Jul 30;12:699078. doi: 10.3389/fmicb.2021.699078 (PMC8363131; doi:10.3389/fmicb.2021.699078)
Supplement: Supplementary file 1 [file Data_Sheet_1.pdf]

Figure S1. The BootScan analysis results for WSMV isolates using the SimPlot program. The recombinants are as follows: (A) NE01_19, (B) COPhil, (C) MON5_20, (D) NS02_19, (E) DC19, (F) KSH294, (G) SM19, (H) KM19, (I) EL17-1183, (J) RO20, and (K) RH20. The reference sequences are found in the legend. The 70% permuted trees support is depicted by the dotted line and is the cut-off support to confirm the potential recombinants.

A

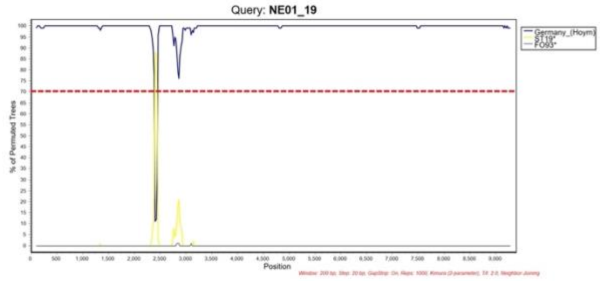

B

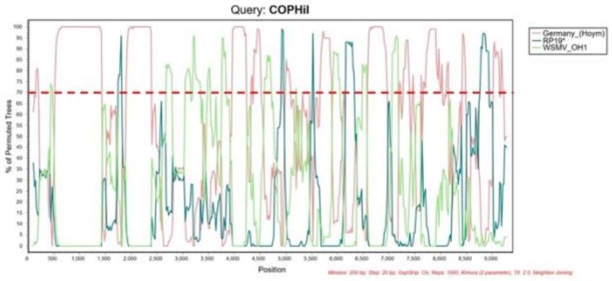

C

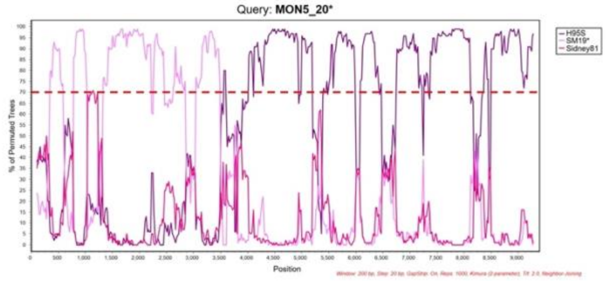

D

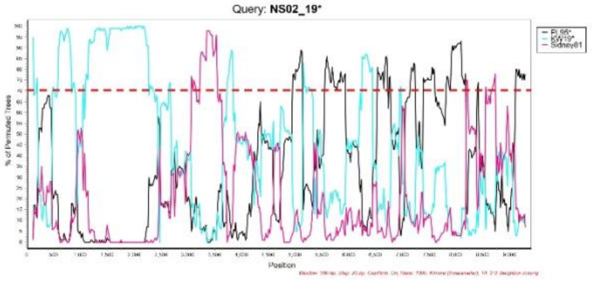

E

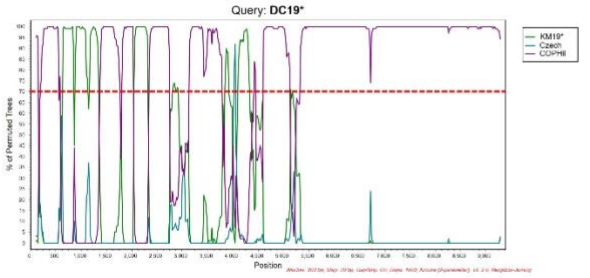

F

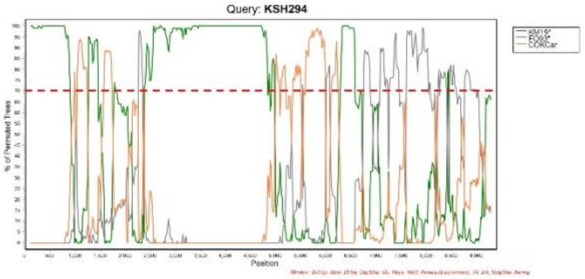

G

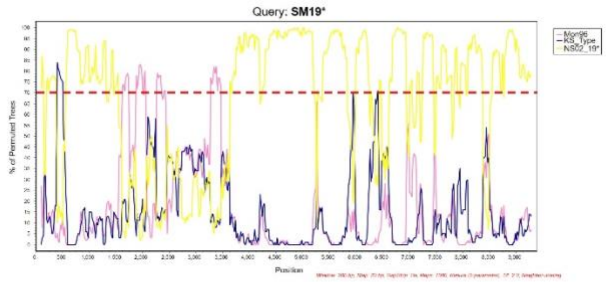

H

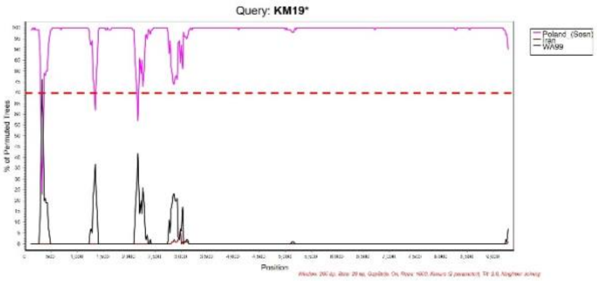

Figure S1 cont.

I

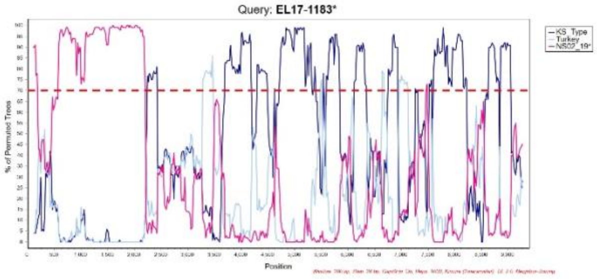

J

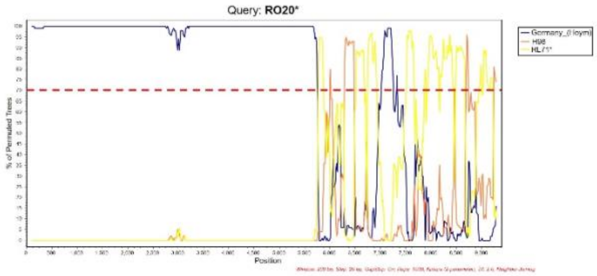

K

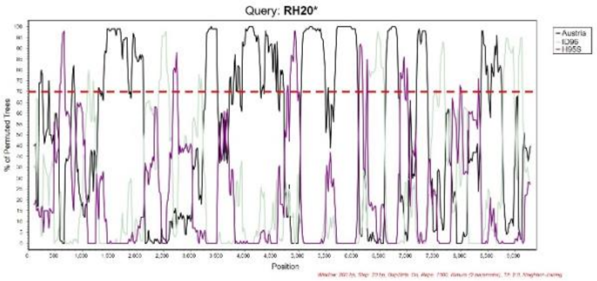

Figure S2. The SimPlot analysis results for WSMV isolates using the SimPlot program. The y-axis shows the nucleotide percent similarity between the query sequence (recombinant) and the reference sequence (major and minor parents shown in the box on the top right). The x-axis depicts the nucleotide position and above this, the schematic of the WSMV genome organization is shown. A crossover between the two references illustrates a recombination breakpoint, shown by the red vertical lines. The recombinants are as follows: NS02_19 (A), DC19 (B), KSH294 (C), SM19 (D), KM19 (E), NE01_19 (F), EL17-1183 (G), RO20 (H), RH20 (I), MON5_20 (J), and COPhil (K).

A

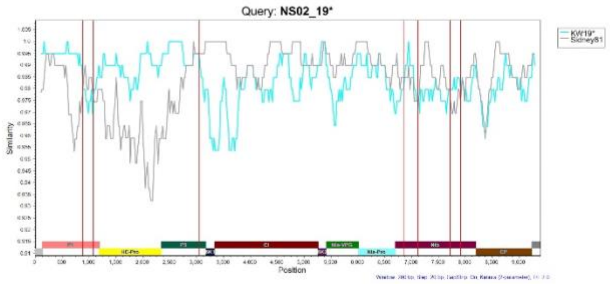

B

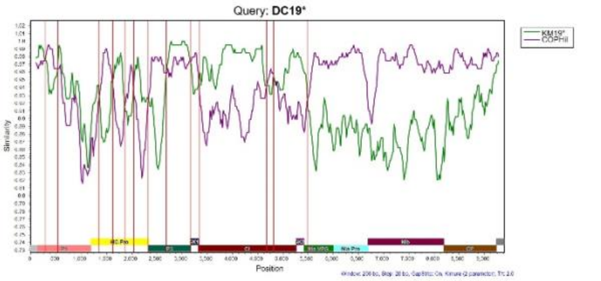

C

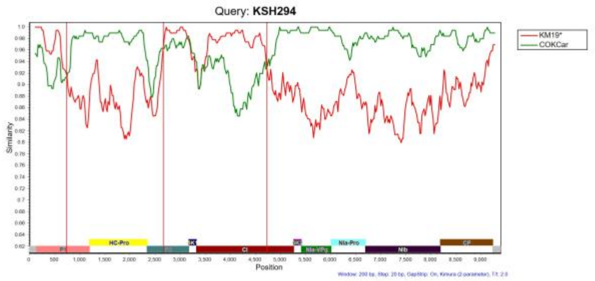

D

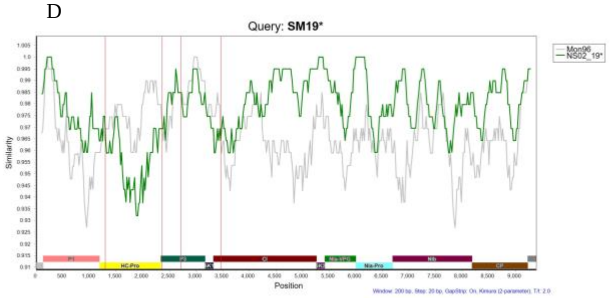

E

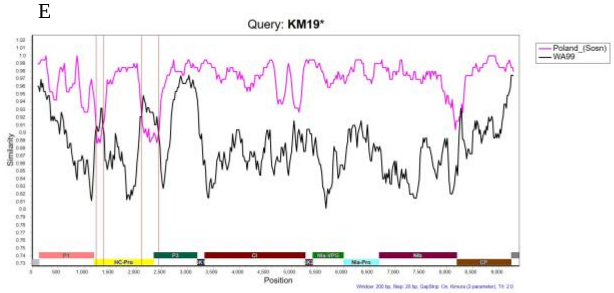

F

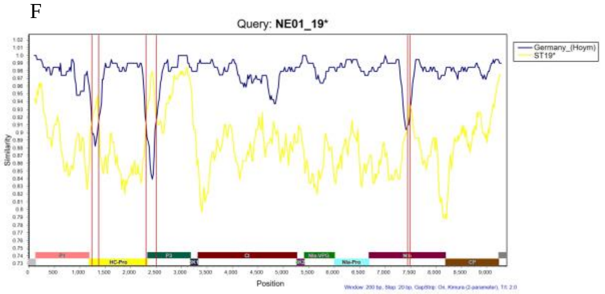

Figure S2 cont.

G

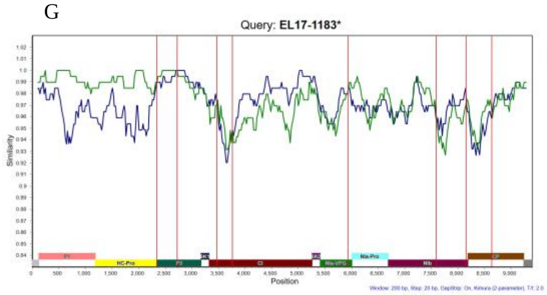

H

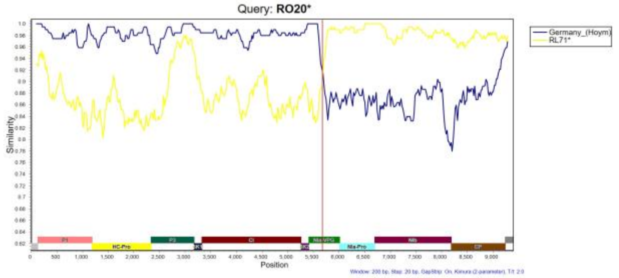

I

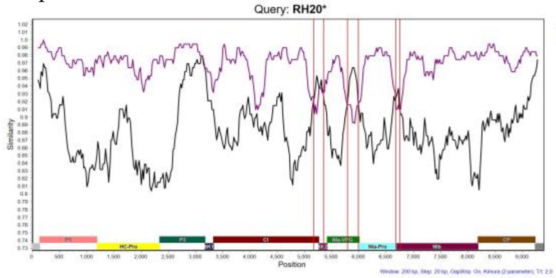

J

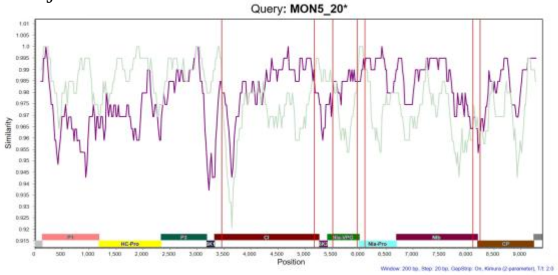

K

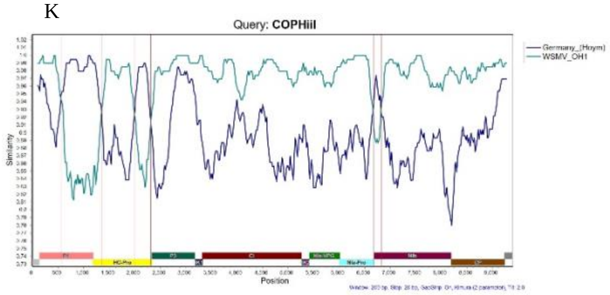

Figure S3. Recombination analysis of TriMV using Bootscan method. Only one recombinant, RH20 was detected. The reference sequences are found in the legend. The 70% permuted trees support is depicted by the dotted line and is the cut-off support to confirm the potential recombinants.

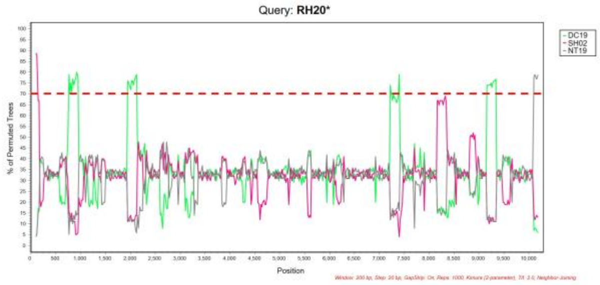

Figure S4. The SimPlot analysis results for a potential recombinant TriMV isolate using the SimPlot program. The y-axis shows the nucleotide percent similarity between the query sequence (recombinant) and the reference sequence (major and minor parents shown in the box on the top right). The x-axis depicts the nucleotide position and above this, the schematic of the TriMV genome organization is shown. A crossover between the two references illustrates a recombination breakpoint, shown by the red vertical lines.

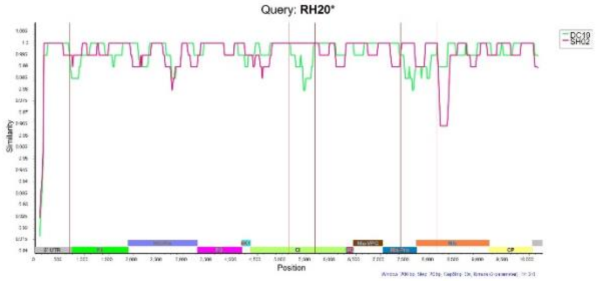

Figure S5. The codon changes in WSMV isolates for positively selected site 118. Isolates with orange branches have shown changes in the P1 from D to N.

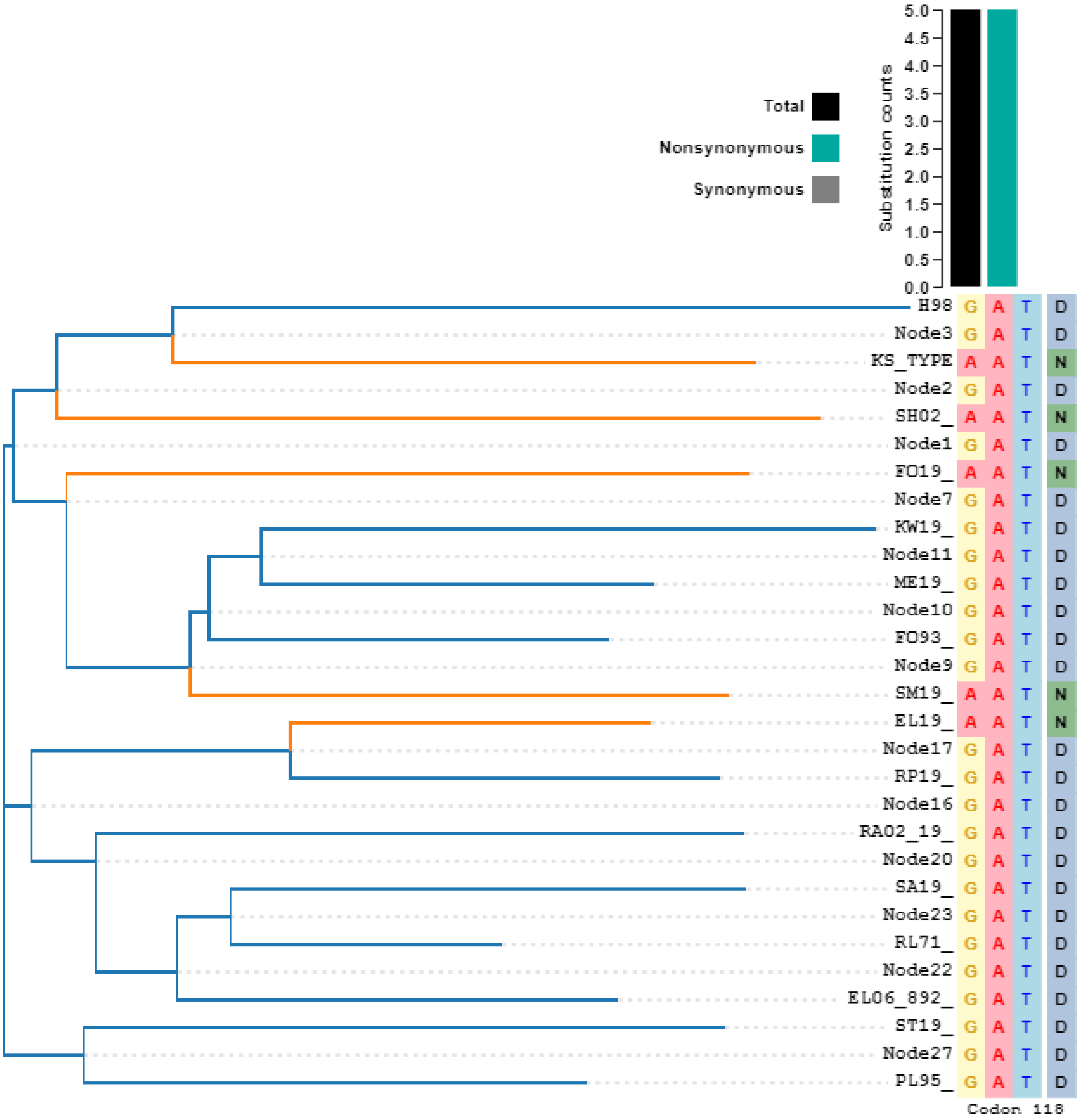

Figure S6. The codon changes in WSMV isolates for positively selected site 2525. Isolates with purple branches have shown changes in the NIb from G to E.

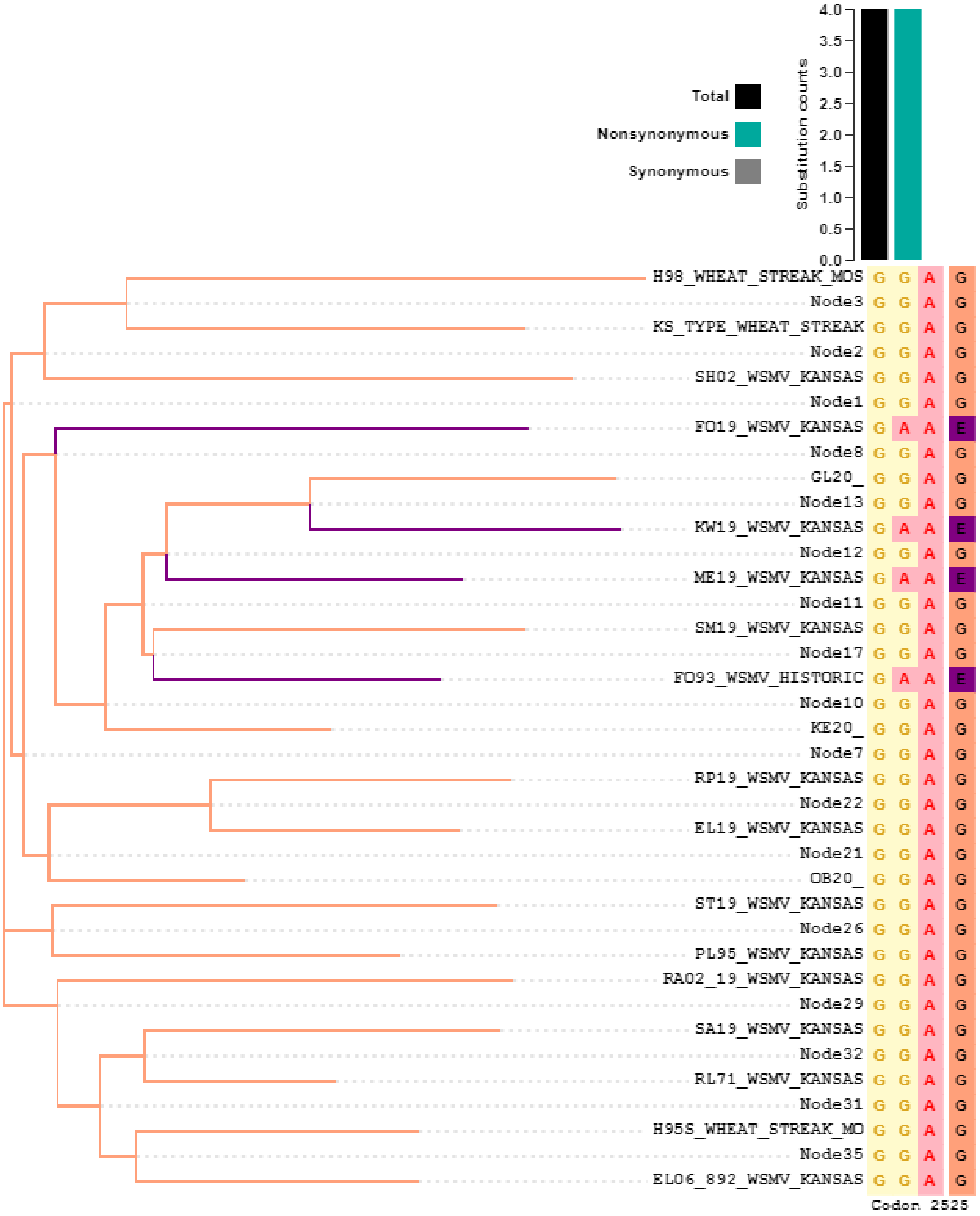

Figure S7. The codon changes in TriMV isolates for positively selected site 2677. RA02_19 isolate with the blue branch showed changes in the NIb from L to I.

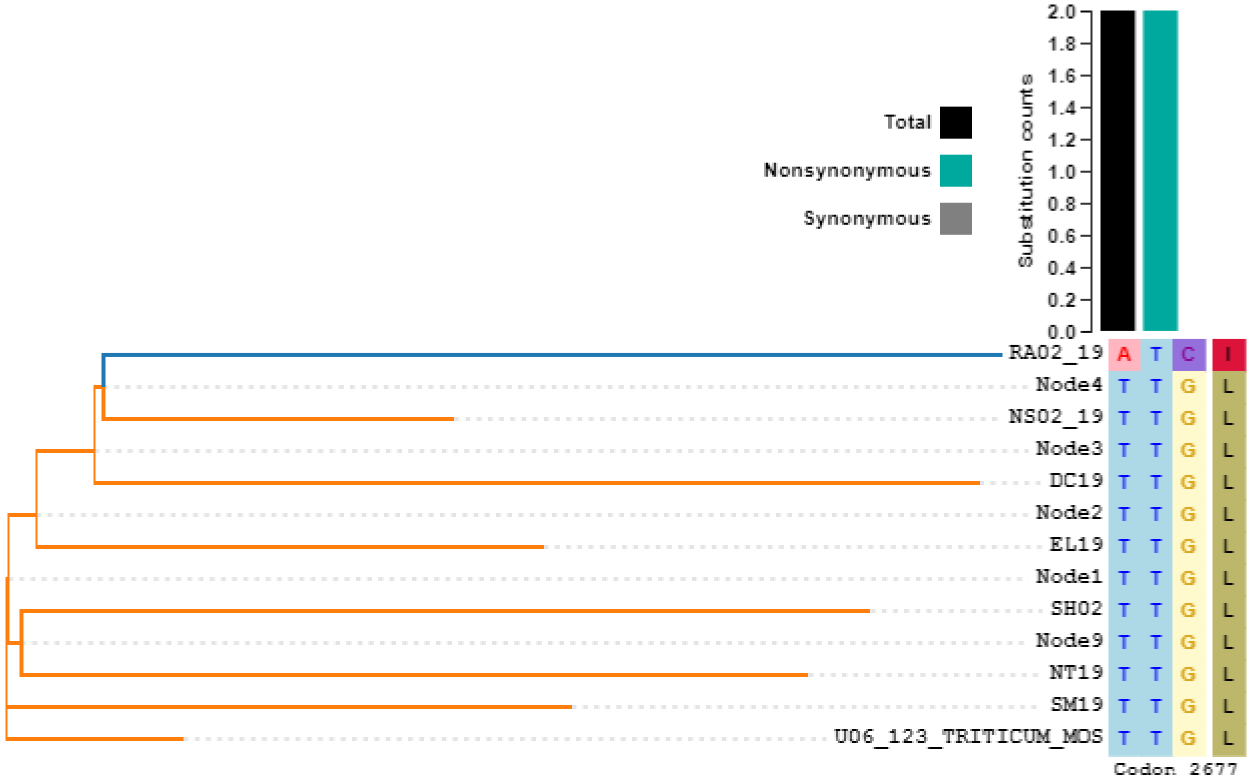

Figure S8. Phylogenetic tree with WSMV recombinants. Below is a highlight of the Clade B, which included only Central Europe isolates. The recombinants from Kansas (KM19 and RO20) and Nebraska (NE01_19) were also grouped within this clade, showing closer relationship to Europe isolates in comparison to other U.S. isolates.

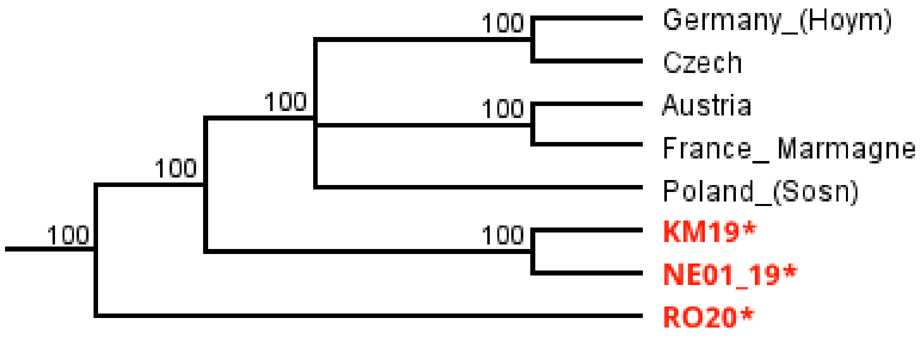

Table S1. List of samples screened for WSM viruses in this study using RT-PCR.

Sample Name	County	Year Collected	WSMV	TriMV	HPWMoV
BA02_19	Barber	2019	-	-	-
BA19	Barber	2019	-	-	-
BT02_19	Barton	2019	+	-	-
BT19	Barton	2019	-	-	-
CK02_19	Cherokee	2019	-	-	-
CK19	Cherokee	2019	-	-	-
CY19	Clay	2019	-	-	-
CM02_19	Comanche	2019	-	-	-
CN02_19	Cheyenne	2019	-	-	-
CN19	Cheyenne	2019	-	-	-
CM19	Comanche	2019	-	-	-
CS19	Chase	2019	-	-	-
DC02_19	Decatur	2019	+	-	-
DC03_19	Decatur	2019	+	-	-
DC19	Decatur	2019	+	+	-
DK01_19	Dickinson	2019	-	-	-
DK02_19	Dickinson	2019	-	-	-
DK03_19	Dickinson	2019	-	-	-
ED19	Edwards	2019	+	-	-
EL19	Ellis	2019	+	+	-
EL06_892	Ellis	2006	+	-	-
EL17_1183	Ellis	2017	+	-	-
EW01_19	Ellsworth	2019	+	-	-
FI02_19	Finney	2019	+	-	-
FI19	Finney	2019	-	-	-
FO19	Ford	2019	+	-	-
FO93	Ford	1993	+	-	-
FW40	Colorado	2019	+	-	-
GH19	Graham	2019	+	-	-
GY19	Gray	2019	+	-	-
GL01_19	Greeley	2019	-	-	-
GL02_19	Greeley	2019	-	-	-
GL03_19	Greeley	2019	-	-	-
HM19	Hamilton	2019	+	-	-
JO19	Johnson	2019	-	-	-
JW02_19	Jewell	2019	+	-	-
JW19	Jewell	2019	+	-	-
KE19	Kearney	2019	+	-	-

Sample Name	County	Year Collected	WSMV	TriMV	HPWMoV
KM19	Kingman	2019	+	-	-
KW19	Kiowa	2019	+	-	-
LE01_19	Lane	2019	+	-	-
LG19	Logan	2019	+	-	-
ME19	Meade	2019	+	-	-
MN01_19	Marion	2019	-	-	-
MC19	Mitchell	2019	+	-	-
MR01_19	Morris	2019	-	-	-
MR02_19	Morris	2019	-	-	-
NE01_19	Nebraska	2019	+	-	-
NS02_19	Ness	2019	+	+	-
NS04_19	Ness	2019	+	-	-
NS19	Ness	2019	+	-	-
NT19	Norton	2019	+	+	-
OB19	Osborne	2019	+	-	-
PL02_19	Phillips	2019	+	-	-
PL95	Phillips	1995	+	-	-
PL19	Phillips	2019	+	-	-
PN02_19	Pawnee	2019	-	-	-
PT19	Pottawatomie	2019	-	-	-
RA02_19	Rawlins	2019	+	+	+
RA19	Rawlins	2019	+	+	-
RL71	Riley	1971	+	-	-
RL05_19	Riley	2019	-	-	-
RL01_19	Riley	2019	-	-	-
RH02_19	Rush	2019	+	-	-
RH19	Rush	2019	+	-	-
ROO2_19	Rooks	2019	+	-	-
RO19	Rooks	2019	+	-	-
RP02_19	Republic	2019	+	-	-
RP19	Republic	2019	+	-	-
RS19	Russell	2019	+	-	-
SA19	Saline	2019	+	-	-
SC01_19	Scott	2019	-	-	-
SC02_19	Scott	2019	+	-	-
SC03_19	Scott	2019	+	-	-
SD19	Sheridan	2019	+	-	-
SG02_19	Sedgwick	2019	-	-	-
SG19	Sedgwick	2019	-	-	-
SH02_19	Sherman	2019	+	+	-
SH19	Sherman	2019	+	-	-

Sample Name	County	Year Collected	WSMV	TriMV	HPWMoV
SM02_19	Smith	2019	+	-	-
SM19	Smith	2019	+	+	-
ST19	Stanton	2019	+	-	-
SU19	Summer	2019	-	-	-
SW19	Seward	2019	+	-	-
TH19	Thomas	2019	+	-	-
TR19	Trego	2019	+	-	-
WA02	Wallace	2019	+	-	-
WA19	Wallace	2019	-	-	-
WH01_19	Wichita	2019	+	-	-
WH02_19	Wichita	2019	-	-	-
WS19	Washington	2019	+	-	-
MON5_20	Montana	2020	+	-	-
RO20	Rooks	2020	+	-	-
OB1_20	Osborne	2020	+	-	-
KM20	Kingman	2020	-	-	-
WH20	Wichita	2020	-	-	-
SA20	Saline	2020	-	-	-
RH20	Rush	2020	+	+	-
KE20	Kearney	2020	+	+	-
GL20	Greeley	2020	+	-	-
RS20	Russell	2020	-	-	-
CM20	Comanche	2020	-	-	-
SA2_20	Saline	2020	-	-	-
EL20	Ellis	2020	-	-	-
NS20	Ness	2020	-	-	-

Table S2. List of all primers used in this study.

Target	Primer Name	Product Size (bp)	Sequences
WSMV	WSMV_Primer3_F	1947	TGAGAAACCACCGAGTTATGACGGC
WSMV	WSMV_Primer3_R		TCCTGGTGCACTGAGAAGTTTGCTG
WSMV	WSMV_Primer5_F	1698	CGAGTGGTTCATTGACGCTGATGGTTC
WSMV	WSMV_Primer5_R		GCCTCTCGTGGAGAAGTACACACCTAG
TriMV	TriMV_Primer3_F	1994	TTGGGAAGCTTCTCAACGAAGGAGAAG
TriMV	TriMV_Primer3_R		TCCGGTAACACTTCCTGAGCCTCGA
TriMV	TriMV_Primer5_F	1953	AGCGAGGGCCATTTATTTGACTGGT
TriMV	TriMV_Primer5_R		AACGTCAGTTTGAAATGCTTGCATGCT
HPWMoV	HPMV_RNA1_F	2006	CAGTCAAACAGGTCCAATGAATCAGACTCT
HPWMoV	HPMV_RNA1_R		AGGGGATATGAGCTAAACAGCACAAAGCT

Table S3. List of samples chosen for library preparation and RNA Sequencing.

Sample Name	County, State	Year Collected	Number of Raw Reads
EL06_892	Ellis, KS	2006	15269574
EL17_1183	Ellis, KS	2017	20475459
EL19	Ellis, KS	2019	15847609
FO19	Ford, KS	2019	17820060
FO93	Ford, KS	1993	20301134
NE01_19	Cheyenne, NE	2019	17111311
NS02_19	Ness, KS	2019	21416060
PL95	Phillips, KS	1995	21855323
RA02_19	Rawlins, KS	2019	15847278
RL71	Riley, KS	1971	19880128
RP19	Republic, KS	2019	15913757
DC19	Decatur, KS	2019	23421520
FW40	CO	2019	28812361
KE19	Kearney, KS	2019	21808767
KM19	Kingman, KS	2019	23649954
KW19	Kiowa, KS	2019	26088658
ME19	Meade, KS	2019	21667049
NT19	Norton, KS	2019	23291475
SA19	Saline, KS	2019	23530569
SH02_19	Sherman, KS	2019	24860180
SM19	Smith, KS	2019	30139534
ST19	Stanton, KS	2019	28329930
MON5_20	Circle, MT	2020	107699365
RO20	Rooks, KS	2020	51059914
OB1_20	Osborne, KS	2020	47162448
RH20	Rush, KS	2020	48713591
KE20	Kearney, KS	2020	84239516
GL20	Greeley, KS	2020	127509132

Table S4. List of all viral reference genomes used for mapping.

Sample ID	Reference Genomes	Accession Number
WSMV	*Wheat streak mosaic virus*	NC_001886.1
TriMV	*Triticum mosaic virus*	NC_012799.1
HPWMoV_RNA1	*High plains mosaic virus*	NC_029570.1
HPWMoV_RNA2	*High plains mosaic virus*	NC_029549.1
HPWMoV_RNA3	*High plains mosaic virus*	NC_029551.1
HPWMoV_RNA4	*High plains mosaic virus*	NC_029551.1
HPWMoV_RNA5	*High plains mosaic virus*	NC_029552.1
HPWMoV_RNA6	*High plains mosaic virus*	NC_029553.1
HPWMoV_RNA7	*High plains mosaic virus*	NC_029554.1
HPWMoV_RNA8	*High plains mosaic virus*	NC_029555.1

Table S5. List of sequences retrieved from the GenBank.

Sample ID	Complete Genome	Country of Origin	Accession Number
El Batan	*Wheat streak mosaic virus*	Mexico	AF285170.1
Austria	*Wheat streak mosaic virus*	Austria	LN624217.1
Marmagne	*Wheat streak mosaic virus*	France	HG810953.1
Sosn	*Wheat streak mosaic virus*	Poland	MH939146.1
Czech	*Wheat streak mosaic virus*	Czech	AF454454.1
Hoym	*Wheat streak mosaic virus*	Germany	HG810954.1
Iran	*Wheat streak mosaic virus*	Iran	EU914917.1
[3]H98	*Wheat streak mosaic virus*	KS, USA	AF511615.2
Mon96	*Wheat streak mosaic virus*	MT, USA	AF511630.2
WA99	*Wheat streak mosaic virus*	WA, USA	AF511643.2
WA94	*Wheat streak mosaic virus*	WA, USA	FJ348358.1
ID96	*Wheat streak mosaic virus*	ID, USA	AF511618.2
Argentina	*Wheat streak mosaic virus*	Argentina	FJ348359.1
Turkey	*Wheat streak mosaic virus*	Turkey	AF454455.1
[3]KS_Type	*Wheat streak mosaic virus*	KS, USA	AF285169.1
KSGre2017	*Wheat streak mosaic virus*	KS, USA	MK318275.1
ID99	*Wheat streak mosaic virus*	ID, USA	AF511619.2
Sidney 81	*Wheat streak mosaic virus*	NE, USA	AF057533.1
H95S	*Wheat streak mosaic virus*	KS, USA	AF511614.2
KSWal2017	*Wheat streak mosaic virus*	KS, USA	MK318281.1
KSHm1	*Wheat streak mosaic virus*	KS, USA	MK318276.1
WSMV_OH1	*Wheat streak mosaic virus*	OH, USA	MK975887.1
[2]KSH294	*Wheat streak mosaic virus*	KS, USA	MF459661.1
[2]COPhil	*Wheat streak mosaic virus*	CO, USA	MT762109.1
COKCar	*Wheat streak mosaic virus*	CO, USA	MT762110.1
[1]ONMV	*Oat necrotic mottle virus*	-	NC_005136.1
[1]YOgMV	*Yellow oat-grass mosaic virus*	-	NC_024471.1
KSGre2017	*Triticum mosaic virus*	KS, USA	MK318272.1
KSHm2015	*Triticum mosaic virus*	KS, USA	MK318273.1
KSIct2017	*Triticum mosaic virus*	KS, USA	MK318274.1
[3]U06-123	*Triticum mosaic virus*	KS, USA	FJ263671.1
NE	*Triticum mosaic virus*	NE, USA	FJ669487.1
COKCar	*Triticum mosaic virus*	CO, USA	MT762125.1
[1]SCSMV	*Sugarcane streak mosaic virus*	-	NC_014037.1
[1]CalVA	*Caladenia virus A*	-	NC_018572.1

[1]These virus isolates were chosen as outgroups for the phylogenetic analysis.
[2] These recombinant isolates were removed from the phylogenetic studies.
[3]These isolates were included in the neutrality test and population genetics analyses.

Table S6. List of the complete viral genome sequences generated in this study.

Sample Name	Complete Genome	County, State	Accession Numbers
[3]RA02_19	*Triticum mosaic virus*	Rawlins, Kansas	MW990193
[3]NS02_19	*Triticum mosaic virus*	Ness, Kansas	MW990200
[3]EL19	*Triticum mosaic virus*	Ellis, Kansas	MW990199
[3]NT19	*Triticum mosaic virus*	Norton, Kansas	MW990196
[3]DC19	*Triticum mosaic virus*	Decatur, Kansas	MW990194
[3]SH02_19	*Triticum mosaic virus*	Sherman, Kansas	MW990195
[2,3]SM19	*Triticum mosaic virus*	Smith, Kansas	MW990198
RH20	*Triticum mosaic virus*	Rush, Kansas	MW990201
KE20	*Triticum mosaic virus*	Kearney, Kansas	MW990197
[3] RA02_19	*Wheat streak mosaic virus*	Rawlins, Kansas	MW990175
[2]NS02_19	*Wheat streak mosaic virus*	Ness, Kansas	MW990189
[3]EL19	*Wheat streak mosaic virus*	Ellis, Kansas	MW990180
[3]RP19	*Wheat streak mosaic virus*	Republic, Kansas	MW990178
[3]FO19	*Wheat streak mosaic virus*	Ford, Kansas	MW990177
[2]NE01_19	*Wheat streak mosaic virus*	Cheyenne, Nebraska	MW990167
[2]DC19	*Wheat streak mosaic virus*	Decatur, Kansas	MW990170
[2]KM19	*Wheat streak mosaic virus*	Kingman, Kansas	MW990168
[3]KW19	*Wheat streak mosaic virus*	Kiowa, Kansas	MW990185
[3]SM19	*Wheat streak mosaic virus*	Smith, Kansas	MW990186
[3]SH02_19	*Wheat streak mosaic virus*	Sherman, Kansas	MW990173
[3]ME19	*Wheat streak mosaic virus*	Meade, Kansas	MW990188
[3]ST19	*Wheat streak mosaic virus*	Stanton, Kansas	MW990174
[3]SA19	*Wheat streak mosaic virus*	Saline, Kansas	MW990179
FW40	*Wheat streak mosaic virus*	Colorado	MW990176
[3]OB20	*Wheat streak mosaic virus*	Osborne, Kansas	MW990192
[2,3]RO20	*Wheat streak mosaic virus*	Rooks, Kansas	MW990169
[2,3]RH20	*Wheat streak mosaic virus*	Rush, Kansas	MW990172
[3]GL20	*Wheat streak mosaic virus*	Greeley, Kansas	MW990182
[3]KE20	*Wheat streak mosaic virus*	Kearney, Kansas	MW990197
MON5_20	*Wheat streak mosaic virus*	Circle, Montana	MW990181
[1,3]PL95	*Wheat streak mosaic virus*	Phillips, Kansas (1995)	MW990184
[1,3]RL71	*Wheat streak mosaic virus*	Riley, Kansas (1971)	MW990190
[1,2]EL17-1183	*Wheat streak mosaic virus*	Ellis, Kansas (2017)	MW990171
[1,3]EL06-892	*Wheat streak mosaic virus*	Ellis, Kansas (2006)	MW990183
[1,3]FO93	*Wheat streak mosaic virus*	Ford, Kansas (1993)	MW990187

[1]These isolates are the historical WSMV samples.
[2]These recombinant isolates were removed from the phylogenetic studies.
[3]These isolates were included in the neutrality test and population genetics analyses.

Table S7. Results from the recombination analysis of WSMV using the RDP5 program.

	RDP5 Methods						
**Recombinants**	**RDP**	**GENECONV**	**BootScan**	**MaxChi**	**Chimaera**	**SiScan**	**3Seq**
**NS02_19**	1.5×10^{-6}	2.5×10^{-3}	9.6×10^{-6}	1.0×10^{-6}	1.0×10^{-6}	2.8×10^{-10}	1.5×10^{-5}
**NE01_19**	1.2×10^{-18}	2.3×10^{-14}	8.7×10^{-19}	7.2×10^{-6}	1.3×10^{-5}	2.5×10^{-3}	1.2×10^{-11}
**DC19**	5.5×10^{13}	2.0×10^{-9}	3.7×10^{-13}	2.2×10^{-4}	2.0×10^{-4}	9.4×10^{-5}	1.4×10^{-6}
**KM19**	7.2×10^{-15}	4.5×10^{-8}	5.6×10^{-15}	9.1×10^{-6}	8.3×10^{-6}	2.9×10^{-2}	1.1×10^{-7}
**EL17-1183**	1.2×10^{-3}	-	1.8×10^{-4}	6.7×10^{-11}	3.9×10^{-5}	1.5×10^{-18}	1.4×10^{-22}
**KSH294**	6.2×10^{-32}	3.4×10^{-27}	4.3×10^{-31}	1.3×10^{-8}	1.7×10^{-9}	1.1×10^{-7}	5.8×10^{-3}
**SM19**	1.2×10^{-2}	-	2.0×10^{-2}	3.7×10^{-4}	7.6×10^{-1}	-	1.7×10^{-4}
**COPhil**	4.7×10^{-56}	5.5×10^{-54}	1.9×10^{-53}	1.9×10^{-17}	6.5×10^{-18}	9.0×10^{-24}	2.9×10^{-35}
**RH20**	4.9×10^{-8}	-	1.9×10^{-3}	3.8×10^{-3}	9.4×10^{-4}	4.2×10^{-3}	1.2×10^{-4}
**RO20**	-	1.9×10^{-66}	4.4×10^{-35}	1.9×10^{-41}	1.1×10^{-6}	1.5×10^{-43}	1.1×10^{-179}
**MON5_20**	4.0×10^{-5}	-	-	3.0×10^{-6}	1.6×10^{-7}	2.7×10^{-2}	6.3×10^{-8}

Table S8. The potential major and minor parents of WSMV recombinants detected by the RDP5 program.

Recombinants	Major Parent	Minor Parent
**NS02**	Sydney 81	KW19
**NE01**	Hoym	ST19
**DC19**	COPhil	KM19
**KM19**	Sosn	WA99
**EL17-1183**	KS_Type	NS02_19
**KSH294**	COKCar	KM19
**SM19**	MON96	NS02_19
**COPhil**	WSMV_OH1	Hoym
**RH20**	H95S	Austria
**RO20**	RL71	Hoym
**MON5_20**	H95S	SM19

Table S9. Results from the recombination analysis of TriMV using the RDP5 program.

	RDP5 Methods						
**Recombinants**	**RDP**	**GENECONV**	**BootScan**	**MaxChi**	**Chimaera**	**SiScan**	**3Seq**
**RH20**	3.3×10^{-4}	3.5×10^{-5}	6.5×10^{-5}	-	1.1×10^{-2}	9.8×10^{-5}	6.4×10^{-3}

Table S10. Full genome sequence of the RNA segments of High plains wheat mosaic emaravirus (HPWMoV).

Sample Name	Complete Genome	County, State	Accession Numbers:
RA02_19	HPWMoV RNA1	Rawlins, Kansas	MW990202
RA02_19	HPWMoV RNA2	Rawlins, Kansas	MW990203
RA02_19	HPWMoV RNA3A	Rawlins, Kansas	MW990204
RA02_19	HPWMoV RNA3B	Rawlins, Kansas	MW990205
RA02_19	HPWMoV RNA4	Rawlins, Kansas	MW990206
RA02_19	HPWMoV RNA5	Rawlins, Kansas	MW990207
RA02_19	HPWMoV RNA6	Rawlins, Kansas	MW990208
RA02_19	HPWMoV RNA7	Rawlins, Kansas	MW990209
RA02_19	HPWMoV RNA8	Rawlins, Kansas	MW990210
